# Translation of Two Healthy Eating and Active Living Support Programs for Parents of 2–6-Year-Old Children: Outcomes of the ‘Time for Healthy Habits’ Parallel Partially Randomised Preference Trial

**DOI:** 10.3390/nu13103348

**Published:** 2021-09-24

**Authors:** Megan L. Hammersley, Rebecca J. Wyse, Rachel A. Jones, Fiona Stacey, Anthony D. Okely, Luke Wolfenden, Marijka J. Batterham, Serene Yoong, Simon Eckermann, Amanda Green, Joe Xu, Christine Innes-Hughes, Jacklyn Jackson, Vincy Li, Chris Rissel

**Affiliations:** 1 Early Start, Faculty of the Arts, Social Sciences and Humanities, University of Wollongong, Wollongong, NSW 2522, Australia; rachelj@uow.edu.au (R.A.J.); tokely@uow.edu.au (A.D.O.); 2 School of Health and Society, Faculty of the Arts, Social Sciences and Humanities, University of Wollongong, Wollongong, NSW 2522, Australia; seckerma@uow.edu.au; 3 Illawarra Health and Medical Research Institute, Wollongong, NSW 2522, Australia; marijka@uow.edu.au; 4 School of Medicine and Public Health, University of Newcastle, University Drive, Callaghan, Newcastle, NSW 2308, Australia; rebecca.wyse@health.nsw.gov.au (R.J.W.); fiona.stacey@health.nsw.gov.au (F.S.); Luke.Wolfenden@health.nsw.gov.au (L.W.); Jacklyn.Jackson@health.nsw.gov.au (J.J.); 5 Hunter New England Population Health, Wallsend, Newcastle, NSW 2287, Australia; 6 Hunter Medical Research Institute, New Lambton Heights, Newcastle, NSW 2305, Australia; 7 Priority Research Centre for Heath Behaviour, University of Newcastle, University Drive, Callaghan, Newcastle, NSW 2308, Australia; 8 School of Education, Faculty of the Arts, Social Sciences and Humanities, University of Wollongong, Wollongong, NSW 2522, Australia; 9 National Institute for Applied Statistics Research Australia, School of Maths and Applied Statistics, Faculty of Engineering and Information Sciences, University of Wollongong, Wollongong, NSW 2522, Australia; 10 School of Health Sciences, Swinburne University of Technology, Hawthorn, Melbourne, VIC 3122, Australia; syoong@swin.edu.au; 11 Centre for Population Health, New South Wales Ministry of Health, St Leonards, Sydney, NSW 2065, Australia; amanda.green@health.nsw.gov.au (A.G.); joe.xu@Health.nsw.gov.au (J.X.); Christine.InnesHughes@health.nsw.gov.au (C.I.-H.); 12 HealthConsult, Sydney, NSW 2000, Australia; vincy.ws.li@gmail.com; 13 College of Medicine and Public Health, Flinders University, Darwin, NT 0800, Australia; chris.rissel@flinders.edu.au

**Keywords:** childhood obesity prevention, fruit, vegetable, intervention, home food environment, healthy eating, screen time, sedentary behaviour, physical activity, movement

## Abstract

This translation study assessed the effectiveness of two remotely delivered healthy eating and active living interventions for parents of 2- to 6-year-old children in improving child fruit and vegetable intake, non-core food intake, body mass index (BMI), physical activity, screen time, and sleep. Parents (*n* = 458) were recruited to a partially randomised preference trial comprising three intervention groups. Healthy Habits Plus comprised six telephone calls, Time2bHealthy comprised six online modules, and the active control comprised ten information sheets and a summary booklet. Data were collected from parents via a telephone questionnaire at baseline and nine months post-baseline. Data were analysed for randomised participants alone (*n* = 240), preference participants alone (*n* = 218), and all participants combined (*n* = 458). There was no significant improvement in fruit and vegetable intake (primary outcome) when comparing the telephone and online interventions to the control. In both the randomised only and all participants combined analyses, there was a significant improvement in non-core food intake for the telephone intervention compared to the control (*p* < 0.001). Differences between interventions for other outcomes were small. In conclusion, the telephone and online interventions did not improve child fruit and vegetable intake relative to written materials, but the telephone intervention did improve non-core food intake.

## 1. Introduction

Establishing positive health behaviours such as healthy dietary intake and movement behaviours (physical activity, sedentary behaviour, and sleep) in early life is critical to reduce the risks of obesity in childhood and beyond [1]. Large-scale population-based childhood obesity prevention and healthy lifestyle programs in New South Wales (NSW), Australia, have concentrated on settings-based and community-level approaches, such as programs delivered in schools and childcare services [2]. Given that parents are critical decision makers and role models, parent-focused interventions have the potential for providing additional gains. Previous research has established that interventions which target parents have more favourable outcomes than those that involve only the child [3,4,5], and it is particularly important to involve parents in interventions focusing on early childhood [4,6].

A range of barriers can impede parent participation in traditional face-to-face interventions [7,8]. Advances in technology have addressed some of these barriers and enabled greater parent engagement. Two interventions which utilise highly accessible mediums have demonstrated promising results in efficacy trials. The Healthy Habits randomised controlled trial (RCT) investigated a 4-week telephone-based intervention delivered to parents of 3- to 5-year-old children. Based on Golan’s family-based model of intervention [9], the intervention specifically targeted fruit and vegetable intake. A significant improvement in child fruit and vegetable intake was found at the 6-month (*p* = 0.021) and 12-month (*p* < 0.01) follow-up [10]. The Time2bHealthy RCT investigated an 11-week online intervention for parents of 2- to 5-year-old children. The intervention focused on healthy eating, physical activity, screen time, and sleep, and was underpinned by Social Cognitive Theory. Significant improvements were found in child discretionary food intake (*p* < 0.01), parent nutrition self-efficacy (*p* = 0.01), and pressure to eat child feeding practices (*p* = 0.048) at the 6-month follow-up [11]. Both interventions were highly acceptable to parents [10,11]. However, these trials were conducted in highly controlled environments and the efficacy of these interventions in the general population was unknown.

Efficacious intervention studies are often not followed up with translation trials, the essential next stage which focuses on intervention effectiveness when they are replicated and scaled up in the community [12]. Translation trials are important to assess whether the results of efficacy trials can be reproduced when offered across a more diverse population using more pragmatic methods of recruitment and implementation [13]. This study, Time for Healthy Habits, expands on the previous RCTs, adapting and scaling these interventions up for implementation across five health jurisdictions (known as local health districts (LHDs)) in NSW, Australia. A parallel partially randomised preference trial design [14,15] was used for this study, selected for its suitability in a real-world setting. This design has two benefits: it allows an assessment of population uptake of interventions of choice, as would occur if these were offered freely to community members, as well as allowing researchers to determine the effect of interventions based on random allocation of participants to an intervention.

The objective of this trial was to determine the effectiveness of the Healthy Habits Plus (telephone) and Time2bHealthy (online) interventions when applied as free services across NSW, Australia, compared to an active control. The evaluation was undertaken for (i) a single intervention in a randomised comparative evaluation and (ii) all interventions in a preference-based evaluation. Respective program uptake was assessed for both of these alternatives.

The primary aims were to: (1) Examine the relative effectiveness of an online behaviour change program (Time2bHealthy), a telephone-based program (Healthy Habits Plus), or a written material active control in targeting parents of 2–6-year-olds in improving child fruit and vegetable consumption (primary outcome). (2) Estimate the relative effectiveness of Time2bHealthy, Healthy Habits Plus, and the active control with respect to improving child consumption of non-core foods, movement behaviours (physical activity, sedentary behaviour (including screen time), and sleep), and weight status in accordance with Australian guidelines (secondary outcomes).

The secondary aim was to determine the preferred ex-ante user delivery medium.

## 2. Materials and Methods

### 2.1. Study Design

The study protocol has been published previously [16]. Briefly, *Time for Healthy Habits* was a translation trial involving parents of 2- to 6-year-old children that sought to improve child fruit and vegetable intake. A parallel partially randomised preference trial design was used. This design initially allowed participants to select their intervention of preference, and once preference trends were established, participants enrolled thereafter were randomised to obtain robust evidence of relative effect. This enabled a comparison between preference-based and randomised uptake and completion rates, as well as post- vs. pre-analysis across all interventions. The trial was conducted between April 2019 and March 2021 across NSW, with five LHDs spanning metropolitan and rural NSW specifically targeted for recruitment. These LHDs have a combined population of over 2.6 million people, and approximately 300,000 children aged 0–9 years. Parent-reported outcome measures were collected at baseline (April 2019 to June 2020), and at 9-month post-baseline follow-up (March 2020 to March 2021) via telephone interview. The Consolidated Standards of Reporting Trials (CONSORT) statement was used to guide reporting of this research [17]. The trial was prospectively registered with the Australian New Zealand Clinical Trials Registry (ACTRN12619000396123p) and was approved by the South Western Sydney LHD Human Research Ethics Committee (HREC) (HE18/300), with site-specific approval granted by Murrumbidgee LHD HREC, Hunter New England LHD HREC, Illawarra Shoalhaven LHD HREC Committee, Southern NSW LHD HREC, and accepted by the University of Wollongong HREC (HE2019/207) and the University of Newcastle HREC (H–2019–0188).

### 2.2. Participant Recruitment and Eligibility Criteria

Recruitment was conducted between April 2019 and April 2020, driven by participating LHDs. Recruitment Officers in each participating LHD conducted face-to-face visits to sites that provided services to young children, including: Early Childhood Education and Care services (childcare services), playgroups, clinics, and other early childhood activities such as library groups. Recruitment Officers promoted and discussed the study and provided parents with information and consent forms. The study was promoted by LHDs to health professionals who were asked to refer appropriate parents to the study. Information and flyers were distributed through local newsletters, community noticeboards, media releases, social media, and local networks. Parents provided informed consent through hard-copy forms or online via the study website.

To be eligible, parents were required to reside in NSW, Australia, have a child aged 2–6 years at baseline, on average live with their child at least four days per week (in order to influence health behaviours), have telephone and internet access (to access the interventions), and speak and understand English. Parents who had previously participated in the Healthy Habits and Time2bHealthy RCTs were excluded.

### 2.3. Randomisation and Blinding

Following consent, participants received a telephone call to confirm eligibility and collect baseline data. Participants were then asked: “Do you have a strong preference for the way in which you receive healthy lifestyle advice or support about your child?” If they responded yes, they were then asked, “Would you prefer to receive healthy lifestyle advice or support via written information, telephone, or online” (with the order in which the interventions were stated randomised). In the initial preference phase of the study, if participants expressed a strong preference for an intervention (online, telephone, or written), they were allocated to that intervention. If they did not express a strong preference, they were randomised to one of the three interventions (using a 1:1:1 ratio). An independent statistician used SAS statistical software (version 9.3, SAS InstituteInc., Cary, NC, USA) to conduct the randomisation. As the main analysis was based on randomised participants only, a stopping rule was applied to allocation based on preference, to ensure that enough participants were randomised. From this point, the trial was conducted as per a traditional randomised controlled trial, with all remaining participants randomised to an intervention (i.e., participants did not have a choice of intervention). This was carried out to provide sufficient power to assess the relative efficacy of the interventions, while enabling relative uptake to be compared between the randomised participants and the preference-based participants.

### 2.4. Interventions

#### 2.4.1. Healthy Habits Plus

Healthy Habits Plus [16] is an enhanced version of the Healthy Habits telephone-based intervention [10]. The revised intervention consisted of six 20- to 30-min telephone support calls intended to be delivered over three months, focusing on healthy eating and movement behaviours (physical activity, screen time, and sleep). Calls were conducted by interviewers with experience in conducting health-related interviews and surveys, but not necessarily with a health qualification. Behaviour change techniques such as barrier identification, goal setting, self-monitoring, prompts, and cues were used during the calls and participants received printed materials to work through in between calls. 

#### 2.4.2. Time2bHealthy

Time2bHealthy is an updated version of the online intervention previously trialled [11], consisting of six modules to be delivered over a 3-month period. Like Healthy Habits Plus, the intervention also focuses on healthy eating and movement behaviours (physical activity, screen time, and sleep). Modules took participants approximately 30 min to complete and included written content, practical activities, videos, and goal setting to facilitate behaviour change. Participants could also join a closed Facebook group to communicate with other participants.

#### 2.4.3. Active Control Group

The active control received written information (total of 10 information sheets and a summary booklet) on current recommendations for child healthy eating and movement behaviours. These materials were developed by the NSW Office of Preventive Health. Participants received these materials via email or post (depending on preference) every fortnight for three months. 

### 2.5. Outcome Measures

#### 2.5.1. Primary Outcome Measures

The primary outcome was child fruit and vegetable intake, assessed using: (i) The fruit and vegetable subscale of the Children’s Dietary Questionnaire (CDQ), which has previously been established as reliable (test–retest intraclass correlation coefficient = 0.75) and valid (Spearman correlation coefficient = 0.58) [18]. (ii) Absolute fruit and vegetable intake serves, using NSW Child Health Survey questions [19]. This was included as the CDQ does not assess number of serves and enabled the absolute change in the number of fruit and vegetable serves consumed daily to be calculated. (iii) Meeting Australian fruit and vegetable guidelines [20], assessed using the serve-based measure data. 

#### 2.5.2. Secondary Outcome Measures

The non-core foods subscale of the CDQ was used to assess child non-core food intake. Child weight status was assessed using World Health Organization (WHO) BMI percentiles [21], calculated using parent-reported responses to child height and weight questions based on the NSW Population health survey (with established reliability and convergent validity) [22,23]. Physical activity and screen time questions were modified from the National Nutrition and Physical Activity Survey [24]. Parents were asked to report how active (in minutes) their child was during the previous day and how much of this activity was of moderate to vigorous intensity. Specifically, they were asked “What was the total time spent doing active play or other physical activity yesterday?” (in hours and minutes), and “In total, how much of this time was moving at a moderate or fast pace (for example, faster walking, running, or jumping?)” (in hours and minutes). Parents were also asked to report the amount of time during the previous day that their child used electronic media devices while sitting or lying down. Parents reported their child’s sleep and nap time (Children’s Sleep Habits Questionnaire [25]). Meeting the Australian 24-h Movement Guidelines for the Early Years [26] was assessed using the physical activity, electronic media, and sleep data. 

### 2.6. Power and Sample Size

Sample size calculations were based on the randomised population only, given that robust relative effects could be shown without confounding across arms in this randomised population. Sample size calculations were based on detecting between-group differences in the proportion of children complying with fruit and vegetable dietary guidelines [18]. Allowing for 20% attrition, 117 randomised participants were required per intervention arm (351 in total) to complete baseline data collection, which would result in a sample size of 93 participants per intervention at the 9-month follow-up post-baseline. For 80% power at a 0.05 significance level, this allowed a 20% detectable difference between the intervention (online and telephone) and control (written) groups in meeting dietary guideline recommendations. The total sample size (636 participants), including preference participants, was based on a more conservative effect size estimate of 15%.

### 2.7. Statistical Analyses

Baseline demographic differences between randomised and preference participants were analysed using independent *t*-tests, Chi Square, and Fishers Exact tests. Outcome analyses were conducted using intention-to-treat principles. The primary outcome of child fruit and vegetable intake was assessed in three ways. The continuous measures, absolute change in (i) fruit and vegetable intake (serves) and (ii) change in CDQ fruit and vegetable subscale score, were analysed using ANCOVA, and the dichotomous measure, (iii) change in meeting the fruit and vegetable guidelines, was analysed using logistic regression. Secondary continuous outcomes (non-core food intake (CDQ non-core subscale), weight status (BMI percentile), physical activity, sedentary screen time, and sleep) were analysed using ANCOVA, and dichotomous outcomes (meeting physical activity, screen time, and sleep guidelines) were analysed using logistic regression.

All analyses were conducted in three ways: (a) complete cases unadjusted, (b) complete cases adjusted for covariates (parent education level, household income, child age, child gender, and child BMI), and (c) multiple imputation for missing data at follow-up, adjusted for covariates. Based on previous studies which have used a similar design [27,28,29], the main analysis was conducted on randomised only participants, with the complete case-adjusted and multiple imputation results in randomised participants being the most robust in avoiding potential for confounding. Separate analyses were also completed for (i) all participants in the dataset, and (ii) preference only participants. Complete case analyses of pooled participants from all interventions were also conducted to explore the whole-of-study effect on the primary outcome from baseline to 9 months post-baseline using paired *t*-tests (absolute change in fruit and vegetable intake and CDQ fruit and vegetable subscale score), and McNemar test (meeting fruit and vegetable guidelines). Significance tests for secondary outcomes were two-tailed with an alpha of 0.05. The alpha level was adjusted to 0.004 for the primary outcome to account for this being measured in three ways (absolute change in fruit and vegetable intake, change in CDQ fruit and vegetable subscale score, and change in meeting fruit and vegetable guidelines).

## 3. Results

### 3.1. Overview

**Figure 1:**
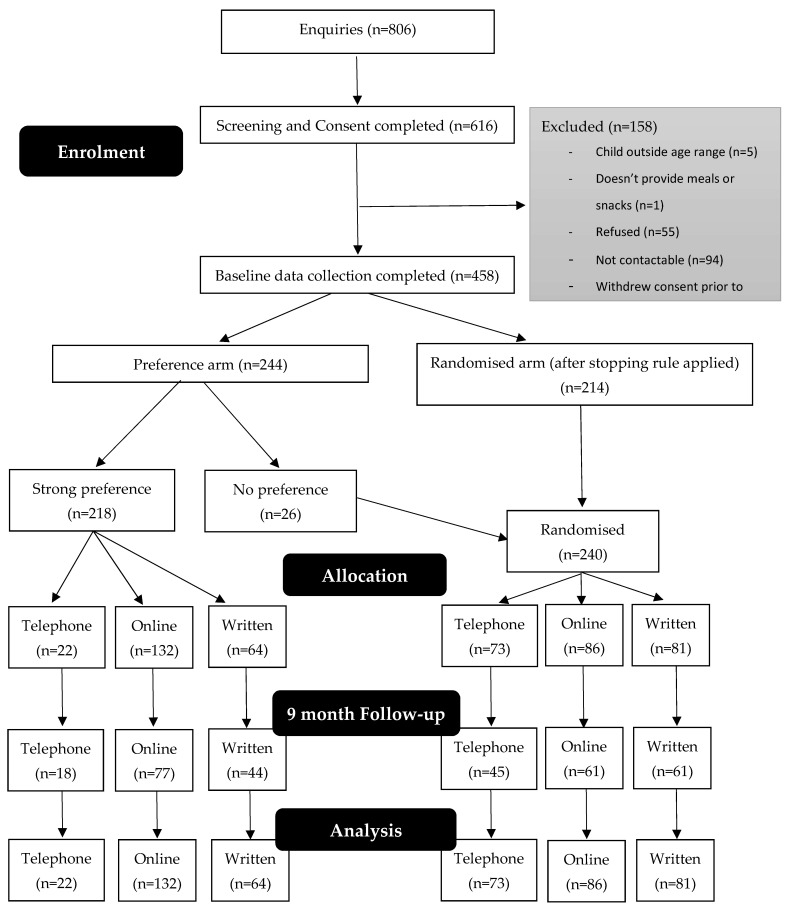
CONSORT flow diagram for the *Time for Healthy Habits* study.

Figure 1 documents the flow of participants through the study. Initial interest was received from 806 parents and 616 completed a consent form. Of these 616 parents, 6 were ineligible, 55 were no longer interested, 94 were not contactable, and 1 withdrew consent at or prior to baseline contact. Baseline data were collected for 458 parents. Prior to the stopping rule being introduced, 244 parents were recruited: 218 expressed a strong preference to intervention and 26 had no preference, so the latter were randomly allocated to an intervention. After application of the stopping rule, 214 participants were recruited, who were all randomly allocated to an intervention. In the preference arm, 22 participants (10%) expressed a strong preference for the telephone intervention, 132 (61%) for the online intervention, and 64 (29%) for the written intervention. In the randomised arm (i.e., those randomised before and after application of the stopping rule), 73 (31%) participants were randomised to the telephone intervention, 86 (36%) to the online intervention, and 81 (34%) to the control. 

Final follow-up (9 months post-baseline) was completed by 306 participants (63 telephone, 138 online, and 105 written). Thirty-two participants actively withdrew from the study, and the main reasons provided were too busy/no time (*n* = 8) or not interested (*n* = 5). An additional 120 participants were lost to follow-up.

**Figure 2:**
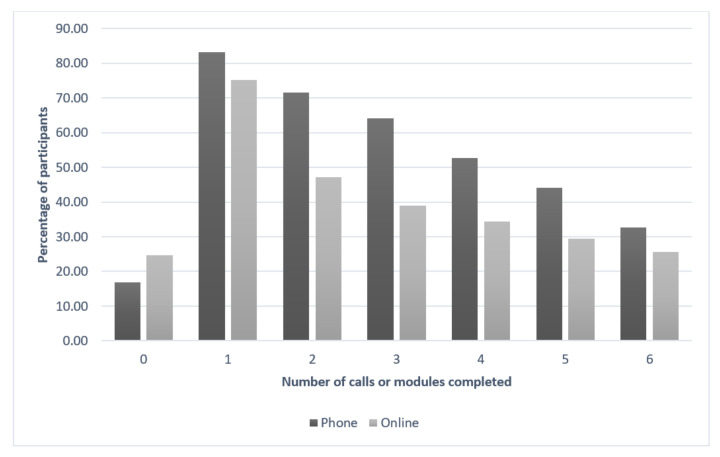
Results of primary outcome (CDQ score, fruit and vegetable daily intake, and fruit and vegetable guideline compliance).

Figure 2 displays the number of telephone calls and online modules completed for the telephone and online interventions. A 20-week timeframe was designated for participants to complete the interventions. There was a higher completion rate for telephone participants compared to online participants overall (33% compared to 26%, respectively), and at every point of the intervention.

### 3.2. Participant Characteristics

Table 1 displays the baseline characteristics of participants. The mean (SD) age of children was 3.37 (1.16) years, 52% were male, and 3.3% were Aboriginal or Torres Strait Islander. The mean (SD) age of parents was 36.12 (4.92) years, and 96% were female, 70% were university qualified, 71% were salary earners, 77% had a household income of over $80,000 per year before tax, and a language other than English was spoken by 18%. There were no significant differences in these demographic characteristics between randomised and preference participants.

### 3.3. Primary Outcome

Table 2 displays the baseline and 9-month post-baseline results for the primary outcome for the randomised participants, preference participants, and the whole sample by intervention, for the complete case-adjusted and multiple imputation analyses. The complete case-unadjusted analyses are provided as a Supplementary Materials (Table S1) as the results did not differ between unadjusted and adjusted analyses.

#### 3.3.1. Primary Outcome Randomised Analyses

There were no significant group-by-time effects on child fruit and vegetable intake (measured by absolute intake, and CDQ fruit and vegetable sub-score) or meeting fruit and vegetable dietary guidelines for any of the interventions compared to the control (written) for the randomised participants.

#### 3.3.2. Primary Outcome Other Analyses

The analyses which included preference participants only, and all participants in the study, found no significant group-by-time effects on child fruit and vegetable intake or meeting dietary guidelines for any of the interventions compared to the control.

The post-hoc pre–post complete case analyses of participants from all interventions (including the control) found that there was a significant improvement in the CDQ fruit and vegetable subscale score, t (306) = −4.233, *p* < 0.0001, fruit and vegetable intake, t (306) = −4.015, *p* < 0.0001, and meeting fruit and vegetable guidelines (*p* < 0.0001). Intake of fruit and vegetable serves increased from a mean of 4.58 (SD = 2.04) serves at baseline to 5.06 (SD = 2.09) serves at 9-month post-baseline, and meeting guidelines increased from 17.8% at baseline to 29.1% at 9-month post-baseline across all interventions.

### 3.4. Secondary Outcomes

Table 3 displays the baseline and 9-month post-baseline results for the secondary analyses for the complete case-adjusted and multiple imputation analyses. The complete case-unadjusted analyses are provided in a Supplementary Materials (Table S2).

#### 3.4.1. Secondary Outcome Randomised Analyses

For the randomised participants, there was a significant group-by-time effect on non-core food intake (non-core CDQ subscale) for the telephone intervention compared to the control (written) based on the multiple imputation analysis only (*p* < 0.001). 

There was a significant difference between the online intervention and the control in relation to change in meeting the screen time guidelines in the opposite direction to that hypothesised based on the adjusted complete case analysis (*p* = 0.008), but this result did not remain significant in the multiple imputation analysis (*p* = 0.063).

#### 3.4.2. Secondary Outcome Other Analyses

Analyses between preference only participants and all participants (i.e., including preference participants) across arms are qualified by potential confounding of participants across their selected intervention. Nevertheless, noting this qualification, there was a significant group-by-time effect on non-core food intake (non-core CDQ subscale) for the telephone intervention (*p* < 0.001) and the online intervention (*p* = 0.038), compared to the control (written) based on the multiple imputation analysis only. For the randomised and preference participants combined, noting this qualification, there was a significant group-by-time effect on non-core food intake (non-core CDQ subscale) for the telephone intervention (*p* < 0.001), compared to the control (written) based on the complete case-adjusted analysis (*p* = 0.040) and when multiple imputation of values was applied (*p* = 0.031).

For preference only participants noting the qualification for potential confounding of relative results between arms, there was also a significant difference between the online intervention and the control in relation to sleep time in the opposite direction to that hypothesised for the complete case-adjusted analysis only (*p* = 0.028). There were no significant results between interventions across analyses including preference participants for any other secondary outcome (BMI percentile, total physical activity, moderate- to vigorous-intensity physical activity, or meeting movement guidelines).

## 4. Discussion

This translation trial of two healthy eating and active living remotely delivered interventions for parents of 2- to 6-year-old children found no significant improvement in child fruit and vegetable intake resulting from participation in either the telephone or online interventions when compared with the active control that received only written information. There was a reduction in child non-core food intake for participants in the telephone intervention, compared to the control. This was the only outcome that was significant among randomised participants, providing high-quality evidence of the impact of the telephone intervention on non-core food consumption. The intervention reduced the mean (SD) CDQ non-core food score from 2.20 (1.04) at baseline, to 1.95 (0.89) at the 9-month follow-up, within the recommended range of ≤2 [18], suggesting a potentially clinically significant change, warranting further investigation. There was also a significant group-by-time interaction for non-core food intake seen in the online intervention when compared to the control in the multiple imputation analysis for the preference participants only, which could be potentially confounded by participants selecting their preferred intervention. These significant secondary outcome results should also be interpreted with caution as no adjustment was made for multiple tests, unlike the primary fruit and vegetable intake outcome which was adjusted to account for the measurement of intake in three different ways. 

Study findings for randomised participants completing the online intervention in relation to meeting screen time guidelines were mixed, with a significantly lower proportion of participants meeting the guidelines nine months post-baseline compared to the control. While this result was significant in the complete cases analyses, it did not remain significant in the multiple imputation analysis and was not found in the original RCT. Potentially, parents may have engaged in the program online content with their children, increasing their child’s level of screen time, or having the unintended effect of negative screen time role modelling. Further research is required to investigate potential unintended effects.

There was also a whole-of-study group effect for fruit and vegetable intake and meeting guidelines in the post-hoc complete cases analyses (with meeting fruit and vegetable guidelines increasing from 18% at baseline to 29% at the 9-month follow-up). Therefore, receiving information via any modality may be enough to enact some amount of change, and provision of written resources could be considered as a minimum strategy. However, these results should be interpreted with caution, as they do not separate intervention effects from secular trends. Additionally, as parents self-selected into this study, it is assumed that they were motivated to obtain support and make changes.

The outcomes of this translation study differ from the original RCTs. Unlike this translation trial, the original Healthy Habits telephone intervention was efficacious in improving fruit and vegetable intake compared to a control [10], but similar to this study, it also demonstrated improvements in non-core food intake. However, in the prior RCT, the non-core food intake results were significant at 2 months, but not at 6 months post-baseline [30]. The original intervention did not include content on movement behaviours, so these outcomes cannot be compared. Unlike the current study, the original Time2bHealthy online intervention RCT demonstrated improvements in discretionary (non-core) food intake compared to the control, at 6 months post-baseline. While there was a significant group-by-time effect for non-core food intake in the preference arm for the online intervention, there was no such effect in the randomised arm. Like the current trial, the prior RCT found no significant improvements in fruit and vegetable intake and movement behaviours compared to the control. 

As the intent of this trial was to deliver the interventions in a similar manner to that implemented in a real-world public health context, less resources were available to ensure engagement with and completion of the intervention content and to invest in ensuring completion of data collection. Given the pragmatic nature of this trial, it is possible that the lower completion rates of the interventions had an impact on effectiveness. In the original Healthy Habits telephone RCT, 87% of participants completed the intervention [10], compared to only 33% in this trial. While there were only four calls in the original intervention, compared to six in the current intervention, only 53% of participants completed four calls in this trial. The Time2bHealthy online RCT completion rate was 26% [11], identical to this trial. No participant incentives were offered in this translation trial, whereas gift cards were provided in the original Time2bHealthy RCT. While this seemingly made no difference to the intervention completion rate, it may have enhanced participation in the final data collection, which was much higher in the prior RCT (96%) compared to this trial (63%). The data collection was face-to-face in the RCT which may have had an influence on attendance as researchers had the chance to develop a rapport. Retention and completion rates have also been reported to be a challenge in other translation trials [31,32,33]. 

The education level of participants in the current trial was generally higher than the original RCTs, with 70% of participants having a university qualification compared to 63% in the Time2bHealthy RCT and 47% in the Healthy Habits RCT, which was not anticipated given the range of demographic characteristics in the areas targeted. Participants with a higher education level may have more baseline knowledge about child healthy eating and movement behaviours, and may have had greater expectations of the interventions, which could account for participants not completing the interventions. Participants receiving only written information may also have been more likely to have the ability to read the materials and capacity to implement the information, which may account for the improvements seen in the written group. Ceiling effects for some outcomes are likely to have reduced the capacity for the trial to detect effects as significant on these measures. For example, a high proportion of participants reported meeting some of the guidelines at baseline, with 79% meeting physical activity guidelines, 97% fruit guidelines, and 84% sleep guidelines. The mean baseline CDQ score was also within the recommended range (≥14), leaving little scope for improvement. This was less the case for meeting screen time and vegetable guidelines, with 48% and 18% meeting guidelines at baseline respectively, although the interventions failed to significantly improve these behaviours. Targeted recruitment of those not meeting guidelines may have facilitated greater relative improvements in these outcomes. Given the high proportion of children meeting fruit guidelines at baseline, focusing on solely vegetable, rather than fruit and vegetable intake, may also be a more effective strategy. A composite score may also be considered in future studies as a combination of small changes in a number of behaviours may have an overall significant positive effect [34]. It is possible that there may have been some recruitment bias, whereby those who had an interest in a healthy lifestyle were more likely to participate. With the high proportion of highly educated participants, there is a need to focus on areas of lower socio-economic status, explore needs, and develop specifically tailored interventions. 

To our knowledge, this is the first parent-focused healthy lifestyle translation trial which focuses on children in the 2- to 6- years’ age group. When interventions are translated, it is common that the effect sizes attenuate relative to those established in efficacy trials [35]. For example, the translation trial of the Go4Fun face-to-face community-based obesity program for children aged 6- to 14-years in NSW found significant improvements in BMI, however improvements in this outcome were half that of the original RCT. Similarly, a study based on the same program (MEND) conducted in the US found improvements in BMI and cardiovascular fitness, but again much lesser improvements were seen compared to the original RCT [36]. Another community-based child obesity program conducted in Queensland, Australia, found a 5–6% improvement in BMI z-score, but similar to Go4Fun, this was much less than the original RCTs (8–10%) [33]. Given the face-to-face nature of these interventions, the different age group, and that these interventions targeted overweight children, it is difficult to make direct comparisons with the current study. 

The study design adopted has not been extensively used, and has been applied primarily in clinical studies, and rarely in public health interventions. For this study, it offered some advantages. Initially allowing participants to choose their preferred intervention supported recruitment efforts as potential participants are often reluctant to participate in studies if there is a chance that they will be randomised to a control group. The study design enabled researchers to identify early in the study participants’ preferred mode of delivery. However, the design is not without its limitations. Due to the high proportion of participants expressing a strong intervention preference in the initial phase, the stopping rule was required, and the study reverted to participants being randomised, which may have negatively affected recruitment efforts thereafter, although this is difficult to ascertain. Lower than anticipated recruitment rates resulted in a randomised sample which was not optimally powered. Before the application of the stopping rule, only 26 of 244 participants (11%) were randomised. The remaining 218 participants (89%) had a strong preference: 132 participants (61%) preferred online, 64 participants (29%) preferred written, and only 22 (10%) preferred telephone. Similar trials, mostly conducted in clinical settings, have generally had a higher proportion of participants opting to be randomised (32–43%) [27,28,29,37,38,39], with the exception of one study where only 3% chose to be randomised [40]. Furthermore, in the preference arm of most of these studies, there has been a more even allocation of participants to the intervention and control groups than in this study. As there seems to be only one other study in a public health setting [39], it is recommended that further research be conducted. In the current trial, after the application of the stopping rule, a question about intervention preference was asked to participants subsequently enrolled, so it would have been an option to collect this information on the whole sample in this manner and conduct an RCT. A cluster RCT design, randomising at the LHD level, may have been a suitable alternative design for estimating relative community and population level effects in practice, although may have presented challenges in matching LHDs in regard to demographics. 

Despite most participants expressing their preference for the online intervention, online completion rates were lower than the telephone intervention (26% for online compared to 33% for telephone by the end of the 20-week timeframe). Furthermore, it was the telephone intervention that demonstrated a significant improvement in non-core food intake compared to the control in the randomised arm. It is possible that while parents initially prefer the convenience of an online intervention that they can complete at any time, their motivation and commitment may have diminished, and they needed more prompts to complete the program other than the email reminders provided. It was largely self-directed, in comparison to the telephone intervention, which was more structured, with the regular telephone calls that were scheduled, and perhaps created a greater sense of accountability, which possibly led to a higher completion rate. The cost of these interventions also needs to be considered. Telephone-based interventions are resource-intensive and expensive, whereas while online interventions may have high set-up costs, they become relatively inexpensive to run on a continuing basis. A cost-effectiveness analysis is therefore planned.

This study was conducted from April 2019 to March 2021, incorporating a period from March to May 2020, when households in NSW first experienced a high level of COVID-19 restrictions. These restrictions required many families to work from home. Schools were closed for all but essential workers’ children, and sporting and other activities ceased operation. Childcare options may have been limited, or parents may have chosen to keep their children home from preschool/childcare. These restrictions hampered face-to-face recruitment efforts during this time and may have impacted on recruitment in other ways as families struggled to balance working from home and child learning supervision responsibilities. COVID-19 may also have impacted on follow-up and program completion rates, which was expressed anecdotally by several participants. It may also have been a factor in cases where participants were not contactable. Several studies have found that COVID-19 restrictions impacted on child food intake and movement behaviours, and it is possible that such impacts may have affected the results of this study [41]. Additionally, immediately prior to the COVID-19 outbreak, NSW endured one of the worst bushfire seasons on record, where fires spanned across large areas of the state. Air quality was impacted in many areas and resulted in people being advised to stay indoors on some days, particularly for those with respiratory conditions [42]. This may have resulted in more limited physical activity opportunities, and greater screen time for children at this time.

Strengths of this study include that parents could participate regardless of location as all interventions were delivered remotely, an important aspect for access and equity, particularly for parents in rural areas, who may have less access to services [43]. The study was partially randomised and had a control group, allowing the opportunity to assess both preference and intervention effects. Unlike some previous translation trials [32,36], an extended follow-up period was included so changes could be assessed long after the completion of the interventions. The study included a range of validated outcome measures which assessed several aspects of dietary intake and movement behaviours. Multiple imputation was used to deal with missing data, with complete case analyses also reported for comparison. These analyses were conducted by an independent statistician. There are several limitations to consider. There were recruitment challenges, with the target number of participants not being achieved. The high drop-out rate and missing covariate data also contributed to a smaller than anticipated sample size. To address this, the analysis also included imputed data using a multiple imputation approach, however multiple imputation introduces extra uncertainty and the smaller than anticipated sample size is a limitation of the study. The study design for preference-based analysis was not fully executed as there were an insufficient number of participants opting to be randomised. The measures were parent-reported, so there may be a degree of social desirability bias, common to most studies that collect self-reported data [44]. Finally, there was a higher proportion of tertiary educated people than in the general population, so the results of this study may not be generalisable to the whole population.

## 5. Conclusions

In this real-world trial, parents preferred online support over telephone-based support, but engaged more with telephone support, and the significant result for improvements in child non-core food intake favoured the telephone intervention. This is a challenge for policymakers in determining the optimal population-level approach that is both acceptable to parents, while achieving the best child healthy lifestyle behaviour outcomes.

## Figures and Tables

**Table 1:** Baseline demographic characteristics of *Time for Healthy Habits* participants by study arm and intervention.

	Randomised			Preference			All
	Control (*n* = 81)	Online (*n* = 86)	Telephone (*n* = 73)	Control (*n* = 64)	Online (*n* = 132)	Telephone (*n* = 22)	(*n* = 458)
	N (%) or Mean (SD)	N (%) or Mean (SD)	N (%) or Mean (SD)	N (%) or Mean (SD)	N (%) or Mean (SD)	N (%) or Mean (SD)	N (%) or Mean (SD)
Child characteristics							
Gender							
-Female	35 (43.2%)	39 (45.3%)	37 (50.7%)	33 (51.6%)	64 (48.5%)	10 (45.5%)	218 (47.6%)
-Male	46 (56.8%)	47 (54.7%)	36 (49.3%)	31 (48.4%)	68 (51.5%)	12 (54.5%)	240 (52.4%)
Age, Mean (SD)	3.25 (1.14)	3.38 (1.14)	3.23 (1.16)	3.73 (1.23)	3.30 (1.15)	3.55 (1.06)	3.37 (1.16)
Aboriginal or Torres Strait Islander origin	3 (3.7%)	4 (4.7%)	1 (1.4%)	3 (4.7%)	3 (2.3%)	1 (4.5%)	15 (3.3%)
Parent characteristics							
Gender							
-Female	79 (97.5%)	83 (96.5%)	69 (94.5%)	62 (96.9%)	126 (95.5%)	22 (100.0%)	441 (96.3%)
-Male	2 (2.5%)	3 (3.5%)	4 (5.5%)	2 (3.1%)	6 (4.5%)	0 (0.0%)	17 (3.7%)
Age, Mean (SD)	36.77 (5.09)	36.58 (4.92)	34.92 (4.52)	35.84 (4.98)	36.09 (4.84)	37.18 (5.49)	36.13 (4.92)
Aboriginal or Torres Strait Islander origin	1 (1.2%)	2 (2.3%)	1 (1.4%)	1 (1.6%)	1 (0.8%)	1 (4.5%)	7 (1.5%)
University or other tertiary education	53 (65.4%)	64 (74.4%)	57 (78.1%)	40 (62.5%)	88 (66.7%)	20 (90.9%)	322 (70.3%)
Employment status in last week							
-Salary earner	62 (76.5%)	55 (64.0%)	53 (72.6)	45 (70.3%)	95 (72.0%)	17 (77.3%)	327 (71.4%)
-Absent on paid leave	8 (9.9%)	5 (5.8%)	3 (4.1%)	6 (9.4%)	14 (10.6%)	2 (9.1%)	38 (8.3%)
-Unpaid work	5 (6.2%)	11 (12.8%)	2 (2.7%)	2 (3.1%)	9 (6.8%)	0 (0.0%)	29 (6.4%)
-Did not have a job	6 (7.4%)	15 (17.4%)	15 (20.5%)	11 (17.2%)	13 (9.8%)	3 (13.6%)	63 (13.8%)
-Don’t know					1 (0.8%)		1 (0.2%)
Annual household income before tax							
Less than $80,000	14 (17.50%)	15 (17.86%)	15 (20.55%)	18 (28.13%)	20 (15.15%)	4 (18.18%)	86 (18.90%)
-$80,000 plus	63 (78.75%)	66 (78.57%)	56 (76.71%)	45 (70.31%)	105 (79.55%)	17 (7.27%)	352 (77.36%)
-Don’t know	3 (3.8%)	3 (3.6%)	2 (2.7%)	1 (1.6%)	7 (5.3%)	1 (4.5%)	17 (3.7%)
Hours in paid work last week	22.54 (17.39)	19.26 (13.53)	28.30 (14.44)	25.77 (15.11)	24.14 (13.83)	21.50 (12.45)	23.70 (14.98)
Language other than English spoken at home	13 (16.0%)	14 (16.3%)	16 (21.9%)	10 (15.6%)	23 (17.4%)	5 (22.7%)	81 (17.7%)

**Table 2:** Results for primary outcome by study group (CDQ score, fruit and vegetable daily intake, and meeting fruit and vegetable guidelines).

		CDQ Total Score				CDQ Total Change Score
		Baseline Mean (SD)	Complete Case ^†^: Mean Difference vs. Control (95% CI)	Complete Case ^†^: Mean Difference vs. Control Adjusted (95% CI)	Multiple Imputation ^‡^: Mean Difference vs. Control (95% CI)	Complete Case ^†^: Mean Difference vs. Control (95% CI)
**Randomised only** (*n* = 240)	Written	Reference	14.51 (5.51)	Reference	Reference	Reference
	Online	0.14 (−1.12, 1.40)	15.68 (3.87)	0.77 (−0.63, 2.16)	0.14 (−1.12, 1.40)	0.14 (–1.12, 1.40)
	Phone	−0.09 (−1.46, 1.27)	16.79 (4.17)	0.76 (−0.83, 2.35)	−0.09 (−1.46, 1.27)	–0.09 (–1.46, 1.27)
**Preference only** (*n* = 218)	Written	Reference	15.75 (3.69)	Reference	Reference	Reference
	Online	0.28 (−1.14, 1.70)	16.36 (4.74)	0.13 (−1.46, 1.73)	0.28 (−1.14, 1.70)	0.28 (–1.14, 1.70)
	Phone	−0.68 (−2.77, 1.42)	14.59 (5.22)	−0.46 (−3.10, 2.19)	−0.68 (−2.77, 1.42)	–0.68 (–2.77, 1.42)
**All** (*n* = 458)	Written	Reference	15.02 (4.85)	Reference	Reference	Reference
	Online	0.17 (−0.75, 1.10)	16.06 (4.79)	0.40 (−0.62, 1.42)	0.17 (−0.75, 1.10)	0.17 (–0.75, 1.10)
	Phone	−0.22 (−1.36, 0.92)	16.16 (4.55)	0.31 (−1.00, 1.63)	−0.22 (−1.36, 0.92)	–0.22 (–1.36, 0.92)
		**Fruit Serves per Day**				
		**Baseline** **Mean (SD)**	**Follow-Up** **Mean (SD)**	**Complete Case ^†^: Mean Difference vs. Control Adjusted (95% CI)**	**Multiple Imputation ^‡^: Mean Difference vs. Control (95% CI)**	
**Randomised only** (*n* = 240)	Written	2.20 (1.02)	2.23 (0.94)	Reference	Reference	
	Online	2.32 (1.20)	2.21 (1.10)	0.03 (−0.31, 0.38)	−0.14 (−0.45, 0.16)	
	Phone	2.22 (0.99)	2.36 (1.05)	0.06 (−0.34, 0.45)	0.03 (−0.32, 0.38)	
**Preference only** (*n* = 218)	Written	2.23 (1.11)	2.57 (0.90)	Reference	Reference	
	Online	2.28 (1.16)	2.36 (1.00)	−0.13 (−0.52, 0.27)	−0.26 (−0.55, 0.02)	
	Phone	2.33 (1.11)	2.11 (0.96)	−0.38 (−1.02, 0.28)	−0.44 (−0.92, 0.04)	
**All** (*n* = 458)	Written	2.21 (1.06)	2.29 (1.03)	Reference	Reference	
	Online	2.29 (1.17)	2.93 (1.04)	−0.11 (−0.43, 0.22)	−0.17 (−0.36, 0.03)	
	Phone	2.25 (1.01)	2.29 (1.03)	−0.05 (−0.30, 0.21)	−0.13 (−0.42, 0.16)	
		**Vegetable Serves per Day**				
		**Baseline** **Mean (SD)**	**Follow-Up** **Mean (SD)**	**Complete Case ^†^: Mean Difference vs. Control Adjusted (95% CI)**	**Multiple Imputation ^‡^: Mean Difference vs. Control (95% CI)**	
**Randomised only** (*n* = 240)	Written	1.56 (0.93)	1.90 (1.08)	Reference	Reference	
	Online	1.68 (1.05)	1.93 (1.06)	−0.14 (−0.52, 0.24)	−0.14 (−0.48, 0.20)	
	Phone	1.81 (0.85)	2.30 (1.16)	0.10 (−0.32, 0.53)	0.20 (−0.21, 0.60)	
**Preference only** (*n* = 218)	Written	1.61 (0.97)	1.82 (1.14)	Reference	Reference	
	Online	1.74 (0.98)	2.11 (1.15)	0.17 (−0.26, 0.61)	−0.03 (−0.39, 0.34)	
	Phone	1.46 (1.01)	1.96 (1.45)	0.10 (−0.61, 0.81)	0.08 (−0.44, 0.58)	
**All** (*n* = 458)	Written	1.59 (0.93)	1.86 (1.10)	Reference	Reference	
	Online	1.71 (1.01)	2.03 (1.11)	0.003 (−0.28, 0.28)	−0.09 (−0.35, 0.18)	
	Phone	1.72 (0.90)	2.20 (1.25)	0.11 (−0.25, 0.46)	0.19 (−0.13, 0.50)	
		**Meeting Fruit and Vegetable Guidelines**				
		**Baseline** **N (%)**	**Follow-Up** **N (%)**	**Complete Case ^†^: Odds vs. Control Adjusted (95% CI)**	**Multiple Imputation ^‡^: Odds vs. Control** **(95% CI)**	
**Randomised only** (*n* = 240)	Written	46/81 (56.8)	38/62 (61.3)	Reference	Reference	
	Online	53/86 (61.6)	46/61 (75.4)	1.53 (0.50, 4.66)	2.04 (0.78, 5.39)	
	Phone	50/73 (68.5)	36/45 (80.0)	1.58 (0.43, 5.86)	2.61 (0.87, 7.79)	
**Preference only** (*n* = 218)	Written	41/64 (64.1)	30/44 (68.2)	Reference	Reference	
	Online	88/132 (66.7)	58/77 (75.3)	1.54 (0.44, 5.40)	1.94 (0.75, 5.04)	
	Phone	11/22 (50.0)	10/18 (55.6)	0.75 (0.10, 5.768)	0.96 (0.21, 4.45)	
**All** (*n* = 458)	Written	87/145 (60.0)	68/106 (64.2)	Reference	Reference	
	Online	141/218 (64.7)	104/138 (75.4)	1.59 (0.71, 3.57)	1.82 (0.93, 3.53)	
	Phone	61/95 (64.2)	46/63 (73.0)	1.28 (0.45, 3.63)	1.94 (0.84, 4.52)	

^†^ Complete case analysis: this analysis included data that reported baseline and follow-up data. ^‡^ Multiple imputations analysis: follow-up data was imputed for missing follow-up and covariate data.

**Table 3:** Results for secondary outcomes by study group (non-core food intake, BMI percentile, total physical activity, moderate to vigorous physical activity, sleep, and meeting physical activity, screen time, and sleep guidelines).

		Non-Core CDQ Score			
		Baseline Mean (SD)	Follow-Up Mean (SD)	Complete Case ^†^: Mean Difference vs. Control Adjusted (95% CI)	Multiple Imputation ^‡^: Mean Difference vs. Control (95% CI)
**Randomised only** (*n* = 240)	Written	2.60 (1.01)	2.47 (1.18)	Reference	Reference
	Online	2.43 (1.18)	2.35 (1.23)	−0.18 (−0.56, 0.20)	−0.05 (−0.13, 0.03)
	Phone	2.20 (1.04)	1.95 (0.89)	−0.33 (−0.75, 0.10)	−0.26 (−0.35, −0.17) *
**Preference only** (*n* = 218)	Written	2.42 (1.13)	2.48 (1.15)	Reference	Reference
	Online	2.29 (1.05)	2.32 (0.97)	0.06 (−0.31, 0.43)	−0.09 (−0.17, −0.01) *
	Phone	2.04 (0.89)	1.83 (0.94)	−0.41 (−1.10, 0.21)	−0.41 (−0.54, −0.28) *
**All** (*n* = 458)	Written	2.55 (1.04)	2.47 (1.16)	Reference	Reference
	Online	2.41 (1.20)	2.33 (1.09)	−0.08 (−0.34, 0.18)	−0.05 (−0.29, 0.18)
	Phone	2.13 (1.03)	1.91 (0.90)	−0.35 (−0.68, −0.02) *	−0.30 (−0.57, −0.03) *
		**BMI Percentile (WHO)**			
		**Baseline** **Mean (SD)**	**Follow-Up** **Mean (SD)**	**Complete Case ^†^: Mean** **Difference vs. Control** **Adjusted (95% CI)**	**Multiple Imputation ^‡^: Mean Difference vs. Control (95% CI)**
**Randomised only** (*n* = 240)	Written	0.65 (0.33)	0.60 (0.32)	Reference	Reference
	Online	0.59 (0.31)	0.60 (0.30)	0.04 (−0.12, 0.19)	0.03 (−0.07, 0.13)
	Phone	0.64 (0.34)	0.64 (0.31)	0.03 (−0.14, 0.20)	0.00 (−0.13, 0.13)
**Preference only** (*n* = 218)	Written	0.59 (0.34)	0.58 (0.32)	Reference	Reference
	Online	0.64 (0.31)	0.55 (0.30)	−0.10 (−0.30, 0.08)	−0.004 (−0.14, 0.13)
	Phone	0.64 (0.35)	0.58 (0.25)	0.04 (−0.20, 0.28)	0.02 (−0.20, 0.23)
**All** (*n* = 458)	Written	0.62 (0.33)	0.59 (0.31)	Reference	Reference
	Online	0.62 (0.31)	0.57 (0.30)	−0.02 (−0.13, 0.09)	0.004 (−0.08, 0.09)
	Phone	0.64 (0.34)	0.62 (0.29)	0.04 (−0.10, 0.18)	0.01 (−0.12, 0.14)
		**Total Physical Activity (Minutes) on Previous Day**			
		**Baseline** **Mean (SD)**	**Follow-Up** **Mean (SD)**	**Complete Case ^†^: Mean** **Difference vs. Control** **Adjusted (95% CI)**	**Multiple Imputation ^‡^: Mean Difference vs. Control (95% CI)**
**Randomised only** (*n* = 240)	Written	317.85 (174.24)	291.33 (134.22)	Reference	Reference
	Online	281.49 (171.31)	226.00 (113.53)	−38.33 (−89.46, 12.81)	−20.89 (−64.91, 23.14)
	Phone	322.92 (149.83)	257.42 (142.54)	20.13 (−37.92, 78.18)	30.70 (−19.93, 81.32)
**Preference only** (*n* = 218)	Written	287.67 (149.40)	275.45 (162.30)	Reference	Reference
	Online	310.00 (169.10)	259.00 (142.31)	−22.72 (−87.02, 41.59)	−24.54 (−78.50, 29.41)
	Phone	293.18 (198.12)	255.00 (177.41)	−61.15 (−166.06, 43.77)	−17.77 (−94.90, 59.35)
**All** (*n* = 458)	Written	304.01 (163.86)	264.91 (150.57)	Reference	Reference
	Online	298.86 (170.14)	244.33 (130.88)	−31.04 (−70.31, 8.23)	−18.48 (−55.07, 18.10)
	Phone	315.86 (161.75)	280.95 (147.27)	−2.95 (−53.68, 47.78)	13.71 (−31.79, 59.20)
		**Moderate to Vigorous Physical Activity (Minutes) on Previous Day**			
		**Baseline** **Mean (SD)**	**Follow-Up** **Mean (SD)**	**Complete Case ^†^: Mean** **Difference vs. Control** **Adjusted (95% CI)**	**Multiple Imputation ^‡^: Mean Difference vs. Control (95% CI)**
**Randomised only** (*n* = 240)	Written	128.28 (95.70)	116.80 (82.76)	Reference	Reference
	Online	131.22 (98.55)	108.39 (78.18)	−12.02 (−40.92, 16.87)	−6.51 (−34.82, 21.80)
	Phone	151.27 (107.74)	134.33 (79.11)	2.47 (−29.94, 34.88)	11.41 (−17.57, 40.39)
**Preference only** (*n* = 218)	Written	124.52 (79.99)	122.84 (81.13)	Reference	Reference
	Online	136.22 (90.45)	112.19 (76.39)	−8.86 (−42.79, 25.06)	−11.06 (−36.30, 14.17)
	Phone	77.36 (60.230)	72.78 (44.27)	−38.58 (−94.63, 17.47)	−30.20 (−71.79, 11.39)
**All** (*n* = 458)	Written	126.64 (88.91)	119.33 (81.74)	Reference	Reference
	Online	134.30 (93.45)	110.49 (76.92)	−10.28 (−31.70, 11.45)	−8.05 (−25.98, 9.88)
	Phone	133.78 (103.24)	116.75 (75.93)	−7.28 (−34.92, 20.36)	1.25 (−22.62, 25.11)
		**Screen Time (Minutes) on Previous Day**			
		**Baseline** **Mean (SD)**	**Follow-Up** **Mean (SD)**	**Complete Case ^†^: Mean** **Difference vs. Control** **Adjusted (95% CI)**	**Multiple Imputation ^‡^: Mean Difference vs. Control (95% CI)**
**Randomised only** (*n* = 240)	Written	112.78 (84.04)	106.45 (105.22)	Reference	Reference
	Online	103.60 (107.11)	114.43 (82.21)	32.18 (−3.40, 67.77)	11.34 (−15.83, 38.51)
	Phone	96.82 (81.69)	88.00 (54.87)	−3.35 (−43.38, 36.69)	−7.83 (−39.00, 23.34)
**Preference only** (*n* = 218)	Written	102.50 (66.72)	115.91 (90.18)	Reference	Reference
	Online	109.60 (80.08)	119.61 (82.40)	0.82 (−37.56, 39.20)	9.51 (−22.36, 41.39)
	Phone	102.62 (82.94)	125.56 (111.14)	36.41 (−26.63, 99.45)	16.91 (−28.54, 62.37)
**All** (*n* = 458)	Written	108.24 (76.80)	110.38 (98.92)	Reference	Reference
	Online	107.23 (91.51)	117.32 (82.06)	17.54 (−8.06, 43.14)	13.29 (−7.13, 33.71)
	Phone	98.13 (81.56)	98.73 (76.23)	4.13 (−28.83, 37.10)	−1.84 (−29.79, 26.10)
		**Sleep (Hours) on Previous Day**			
		**Baseline** **Mean (SD)**	**Follow-Up** **Mean (SD)**	**Complete Case ^†^: Mean** **Difference vs. Control** **Adjusted (95% CI)**	**Multiple Imputation ^‡^: Mean Difference vs. Control (95% CI)**
**Randomised only** (*n* = 240)	Written	11.66 (1.13)	11.23 (0.91)	Reference	Reference
	Online	11.32 (1.02)	11.08 (0.86)	−0.01 (−0.36, 0.34)	−0.10 (−0.43, 0.23)
	Phone	11.38 (1.19)	11.03 (1.01)	0.03 (−0.37, 0.43)	−0.10 (−0.43, 0.23)
**Preference only** (*n* = 218)	Written	11.46 (1.02)	11.15 (0.96)	Reference	Reference
	Online	11.50 (1.08)	10.88 (1.00)	−0.48 (−0.91, −0.05) *	−0.29 (−0.58, 0.18)
	Phone	11.14 (1.44)	11.14 (0.80)	0.07 (−0.64, 0.78)	0.15 (−0.38, 0.69)
**All** (*n* = 458)	Written	11.57 (1.09)	11.20 (0.93)	Reference	Reference
	Online	11.43 (1.06)	10.97 (0.95)	−0.24 (−0.51, 0.03)	−0.17 (−0.41, 0.07)
	Phone	11.32 (1.25)	11.06 (0.95)	−0.02 (−0.37, 0.33)	−0.05 (−0.35, 0.26)
		**Meeting Physical Activity Guidelines**			
		**Baseline** **N (%)**	**Follow-Up** **N (%)**	**Complete Case ^†^: Odds vs. Control Adjusted (95% CI)**	**Multiple Imputation ^‡^: Odds vs. control (95% CI)**
**Randomised only** (*n* = 240)	Written	62/79 (78.5)	45/61 (73.8)	Reference	Reference
	Online	62/82 (75.6)	41/59 (67.2)	0.68 (0.25, 1.91)	0.75 (0.29, 1.97)
	Phone	62/72 (86.1)	37/45 (82.2)	2.06 (0.59, 7.20)	1.24 (0.48, 3.24)
**Preference only** (*n* = 218)	Written	47/61 (77.0)	35/44 (79.5)	Reference	Reference
	Online	105/131 (80.2)	51/73 (66.2)	0.38 (0.12, 1.24)	0.46 (0.19, 1.08)
	Phone	15/22 (68.2)	11/18 (61.1)	0.19 (0.03, 1.24)	0.42 (0.11, 1.57)
**All** (*n* = 458)	Written	109/140 (77.9)	80/105 (76.2)	Reference	Reference
	Online	167/213 (78.4)	92/132 (69.7)	0.58 (0.28, 1.20)	0.66 (0.36, 1.21)
	Phone	77/94 (81.9)	48/63 (76.2)	0.99 (0.38, 2.57)	0.93 (0.45, 1.92)
		**Meeting Screen Time Guidelines**			
		**Baseline** **N (%)**	**Follow-Up** **N (%)**	**Complete Case ^†^: Odds vs. Control Adjusted (95% CI)**	**Multiple Imputation ^‡^: Odds vs. Control (95% CI)**
**Randomised only** (*n* = 240)	Written	35/81 (43.2)	29/62 (46.8)	Reference	Reference
	Online	43/86 (50.0)	22/61 (36.1)	0.25 (0.09, 0.69)*	0.43 (0.17, 1.05)
	Phone	41/72 (56.9)	25/45 (55.6)	0.82 (0.28, 2.41)	1.31 (0.58, 2.99)
**Preference only** (*n* = 218)	Written	30/64 (46.9)	20/44 (45.5)	Reference	Reference
	Online	62/132 (47.0)	33/77 (42.9)	1.02 (0.39, 2.7)	0.73 (0.32, 1.68)
	Phone	10/21 (47.6)	10/18 (55.6)	1.11 (0.23, 5.47)	1.40 (0.44, 0.47)
**All** (*n* = 458)	Written	65/145 (44.8)	49/106 (46.2)	Reference	Reference
	Online	105/218 (48.2)	55/138 (39.9)	0.56 (0.29, 1.09)	0.60 (0.32, 1.11)
	Phone	51/93 (54.8)	35/63 (55.6)	1.02 (0.44, 2.38)	1.34 (0.67, 2.70)
		**Meeting Sleep Guidelines**			
		**Baseline** **N (%)**	**Follow-Up** **N (%)**	**Complete Case ^†^: Odds vs. Control Adjusted (95% CI)**	**Multiple Imputation ^‡^: Odds vs. Control (95% CI)**
**Randomised only** (*n* = 240)	Written	70/81 (86.4)	53/62 (85.5)	Reference	Reference
	Online	73/86 (84.9)	55/61 (90.2)	2.12 (0.46, 9.67)	1.66 (0.52, 5.31)
	Phone	58/73 (79.5)	33/45 (73.3)	0.89 (0.22, 3.60)	0.64 (0.24, 1.73)
**Preference only** (*n* = 218)	Written	54/64 (84.4)	37/42 (88.1)	Reference	Reference
	Online	114/132 (86.4)	62/77 (80.5)	0.37 (0.08, 1.79)	0.64 (0.24, 1.84)
	Phone	16/22 (72.7)	14/18 (77.8)	1.82 (0.14, 23.12)	0.64 (0.12, 3.32)
**All** (*n* = 458)	Written	124/145 (85.5)	90/104 (86.5)	Reference	Reference
	Online	187/218 (85.8)	117/138 (84.8)	0.78 (0.30, 2.04)	0.90 (0.44, 1.86)
	Phone	74/95 (77.9)	47/63 (74.6)	0.99 (0.31, 3.17)	0.60 (0.26, 1.34)

^†^ Complete case analysis: this analysis included data that reported baseline and follow-up data. ^‡^ Multiple imputations analysis: follow-up data was imputed for missing follow-up and covariate data. * *p* < 0.05.

## Data Availability

The data are not publicly available as data sharing was not sought in the ethics application. Therefore ethics approval has been obtained for data to be used for this study only.

## Supplementary Materials

The following are available online at https://www.mdpi.com/article/10.3390/nu13103348/s1, Table S1: Results for primary outcome by study group (CDQ score, fruit and vegetable daily intake, and meeting fruit and vegetable guidelines) complete case-unadjusted results. Table S2: Results for secondary outcomes by study group (non-core food intake, BMI percentile, total physical activity, moderate to vigorous physical activity, sleep, and compliance with physical activity and sleep guidelines) complete case analysis, unadjusted.
