# Supplementary material for: Translation of Two Healthy Eating and Active Living Support Programs for Parents of 2–6-Year-Old Children: Outcomes of the ‘Time for Healthy Habits’ Parallel Partially Randomised Preference Trial
Source: Nutrients. 2021 Sep 24;13(10):3348. doi: 10.3390/nu13103348 (PMC8539933; doi:10.3390/nu13103348)
Supplement: Supplementary file 1 [file nutrients-13-03348-s001.zip › nutrients-1391701-supplementary.pdf]

Table S1. Results for primary outcome by study group (CDQ Score, Fruit and Vegetable Daily Intake, and Meeting Fruit and Vegetable Guidelines) complete case unadjusted results.

		**CDQ Total score**
		Complete case†:<br>Mean difference vs control (95% CI)
**RANDOMISED ONLY** (n=240)	Written	Reference
	Online	0.42 (-0.75, 1.58)
	Phone	0.55 (-0.73,1.83)
**PREFERENCE ONLY** (n=218)	Written	Reference
	Online	0.40 (-0.88,1.68)
	Phone	-0.85 (-2.74,1.04)
**ALL** (n=458)	Written	Reference
	Online	0.43 (-0.41,1.28)
	Phone	0.19 (-0.85,1.24)
		**Fruit Serves per Day**
		Complete case†:<br>Mean difference vs control (95% CI)
**RANDOMISED ONLY** (n=240)	Written	Reference
	Online	-0.09 (-0.39,0.21)
	Phone	0.16 (-0.17,0.48)
**PREFERENCE ONLY** (n=218)	Written	Reference
	Online	-0.21 (-0.53,0.11)
	Phone	-0.46 (-0.93,0.01)
**ALL** (n=458)	Written	Reference
	Online	-0.11 (-0.33,0.10)
	Phone	-0.06 (-0.33,0.20)
		**Vegetable Serves per Day**
		Complete case†:<br>Mean difference vs control (95% CI)
**RANDOMISED ONLY** (n=240)	Written	Reference
	Online	-0.14 (-0.46,0.18)
	Phone	0.17 (-0.18,0.51)
**PREFERENCE ONLY** (n=218)	Written	Reference
	Online	0.10 (-0.27,0.45)
	Phone	0.16 (-0.36,0.65)
**ALL** (n=458)	Written	Reference
	Online	-0.36 (-0.27,0.20)
	Phone	0.18 (-0.10,0.46)
		**Meeting Fruit and Vegetable Guidelines**
		Complete case†:<br>Odds vs control<br>(95% CI)
**RANDOMISED ONLY** (n=240)	Written	Reference
	Online	1.92 (0.74,4.99)
	Phone	2.51 (0.85,7.40)
**PREFERENCE ONLY** (n=218)	Written	Reference
	Online	1.64 (0.63,4.25)
	Phone	0.70 (0.19,2.64)
**ALL** (n=458)	Written	Reference
	Online	1.77 (0.91,3.44)
	Phone	1.63 (0.72,3.68)

†Complete case analysis: this analysis included data that reported baseline and follow-up data.

Table S2: Results for secondary outcomes by study group (non-core food intake, BMI percentile, total physical activity, moderate to vigorous physical activity, sleep, and compliance with physical activity and sleep guidelines) complete case analysis unadjusted.

		Non-core CDQ Score
		Complete case†:<br>Mean difference vs control (95% CI)
**RANDOMISED ONLY** (n=240)	Written	Reference
	Online	-0.02 (-0.34,0.29)
	Phone	-0.22 (-0.57,0.13)
**PREFERENCE ONLY** (n=218)	Written	Reference
	Online	-0.11 (-0.43,0.21)
	Phone	-0.47 (-0.95,0.01)
**ALL** (n=458)	Written	Reference
	Online	-0.06 (-0.28,0.17)
	Phone	-0.32 (-0.59,-0.04)*
		**BMI Percentile (WHO)**
		Complete case†:<br>Mean difference vs control (95% CI)
**RANDOMISED ONLY** (n=240)	Written	Reference
	Online	0.07 (-0.08,0.21)
	Phone	0.05 (-0.11,0.22)
**PREFERENCE ONLY** (n=218)	Written	Reference
	Online	-0.06 (-0.23,0.10)
	Phone	0.02 (-0.21,0.25)
**ALL** (n=458)	Written	Reference
	Online	-0.01(-0.11,0.10)
	Phone	0.04 (-0.09,0.18)
		**Total Physical Activity (minutes) on Previous Day**
		Complete case†:<br>Mean difference vs control (95% CI)
**RANDOMISED ONLY** (n=240)	Written	Reference
	Online	-19.67 (-76.15,36.80)
	Phone	-20.59 (-103.73,62.55)
**PREFERENCE ONLY** (n=218)	Written	Reference
	Online	-27.55 (-71.92,16.82)
	Phone	29.36 (-18.30,77.02)
**ALL** (n=458)	Written	Reference
	Online	-20.53 (-55.36,14.30)
	Phone	12.13 (-30.57,54.83)
		**Moderate to Vigorous Physical Activity (minutes) on Previous Day**
		Complete case†:<br>Mean difference vs control (95% CI)
**RANDOMISED ONLY** (n=240)	Written	Reference
	Online	-7.13 (-34.86,20.60)
	Phone	10.19 (-19.54,39.91)
**PREFERENCE ONLY** (n=218)	Written	Reference
	Online	-11.60 (-39.58,16.39)
	Phone	-37.89 (-79.59,3.82)
**ALL** (n=458)	Written	Reference
	Online	-9.52 (-28.97,9.94)
	Phone	-2.52(-26.17,21.09
		**Screen Time (minutes) on Previous Day**
		Complete case†:<br>Mean difference vs control (95% CI)
**RANDOMISED ONLY** (n=240)	Written	Reference
	Online	11.27 (-17.78,40.31)
	Phone	-16.05 (-47.57,15.47)
**PREFERENCE ONLY** (n=218)	Written	Reference
	Online	-1.22 (-31.75,29.30)
	Phone	15.99 (-30.04,62.02)
**ALL** (n=458)	Written	Reference
	Online	7.07 (-13.72,27.87)
	Phone	-8.64 (-34.38,17.11)
		**Sleep (hours) on Previous Day**

		Complete case[†]: Mean difference vs control (95% CI)
**RANDOMISED ONLY** (n=240)	Written	Reference
	Online	-0.03 (-0.33,0.26)
	Phone	-0.12 (-0.44,0.20)
**PREFERENCE ONLY** (n=218)	Written	Reference
	Online	-0.26 (-0.63,0.10)
	Phone	0.07 (-0.48,0.61)
**ALL** (n=458)	Written	Reference
	Online	-0.18 (-0.41,0.05)
	Phone	-0.05 (-0.34,0.23)
		**Meeting Physical Activity Guidelines**
		Complete case[†]: Odds vs control (95% CI)
**RANDOMISED ONLY** (n=240)	Written	Reference
	Online	0.88 (0.38,2.01)
	Phone	1.47 (0.55,3.90)
**PREFERENCE ONLY** (n=218)	Written	Reference
	Online	0.42 (0.12,1.53)
	Phone	0.57 (0.22,1.47)
**ALL** (n=458)	Written	Reference
	Online	0.74 (0.40,1.36)
	Phone	0.91 (0.43,1.96)
		**Meeting Screen Time Guidelines**
		Complete case[†]: Odds vs control (95% CI)
**RANDOMISED ONLY** (n=240)	Written	Reference
	Online	0.42 (0.18,0.96)
	Phone	1.22 (0.518,2.883)
**PREFERENCE ONLY** (n=218)	Written	Reference
	Online	0.85 (0.39,1.87)
	Phone	1.29 (0.40,4.15)
**ALL** (n=458)	Written	Reference
	Online	0.64 (0.37,1.11)
	Phone	1.27 (0.65,2.50)
		**Meeting Sleep Guidelines**
		Complete case[†]: Odds vs control (95% CI)
**RANDOMISED ONLY** (n=240)	Written	Reference
	Online	1.63 (0.54,4.93)
	Phone	0.50 (0.19,1.32)
**PREFERENCE ONLY** (n=218)	Written	Reference
	Online	0.49 (0.16,1.56)
	Phone	0.50 (0.11,2.33)
**ALL** (n=458)	Written	Reference
	Online	0.88 (0.42,1.85)
	Phone	0.50 (0.22,1.13)

†Complete case analysis: this analysis included data that reported baseline and follow-up data.
‡ Multiple imputations analysis: follow-up data was imputed for missing follow-up and covariate data.
* p <0.05,
